# Plasticity of white matter connectivity in phonetics experts

**DOI:** 10.1007/s00429-015-1114-8

**Published:** 2015-09-19

**Authors:** Maaike Vandermosten, Cathy J. Price, Narly Golestani

**Affiliations:** 1 Parenting and Special Education Research Unit, KU Leuven, Leuven, Belgium; 2 Wellcome Trust Centre for Neuroimaging, Institute of Neurology, University College London, London, UK; 3 Brain and Language Lab, Department of Clinical Neuroscience, Campus Biotech, University of Geneva, 9 Chemin des Mines, 1202 Geneva, Switzerland

**Keywords:** White matter, Plasticity, Phonetics, Expertise, Auditory cortex, Broca’s area

## Abstract

Phonetics experts are highly trained to analyze and transcribe speech, both with respect to faster changing, phonetic features, and to more slowly changing, prosodic features. Previously we reported that, compared to non-phoneticians, phoneticians had greater local brain volume in bilateral auditory cortices and the left pars opercularis of Broca’s area, with training-related differences in the grey-matter volume of the left pars opercularis in the phoneticians group (Golestani et al. 2011). In the present study, we used diffusion MRI to examine white matter microstructure, indexed by fractional anisotropy, in (1) the long segment of arcuate fasciculus (AF_long), which is a well-known language tract that connects Broca’s area, including left pars opercularis, to the temporal cortex, and in (2) the fibers arising from the auditory cortices. Most of these auditory fibers belong to three validated language tracts, namely to the AF_long, the posterior segment of the arcuate fasciculus and the middle longitudinal fasciculus. We found training-related differences in phoneticians in left AF_long, as well as group differences relative to non-experts in the auditory fibers (including the auditory fibers belonging to the left AF_long). Taken together, the results of both studies suggest that grey matter structural plasticity arising from phonetic transcription training in Broca’s area is accompanied by changes to the white matter fibers connecting this very region to the temporal cortex. Our findings suggest expertise-related changes in white matter fibers connecting fronto-temporal functional hubs that are important for phonetic processing. Further studies can pursue this hypothesis by examining the dynamics of these expertise related grey and white matter changes as they arise during phonetic training.

**Electronic supplementary material:**

The online version of this article (doi:10.1007/s00429-015-1114-8) contains supplementary material, which is available to authorized users.

## Introduction

A growing number of studies show brain structural differences in grey (Richardson and Price 2009; Mechelli et al. 2004; Elmer et al. 2013; Bermudez et al. 2009) and white matter (Roberts et al. 2013; Elmer et al. 2011; Bengtsson et al. 2005) between expert and non-expert individuals, in linguistic, musical, and other domains (Golestani 2014). These differences have been attributed to training-related plasticity (Steele et al. 2013; Zatorre et al. 2012; Klein et al. 2014; Imfeld et al. 2009; Tavor et al. 2013; Sampaio-Baptista et al. 2013; Schlegel et al. 2012; Draganski et al. 2014; Seither-Preisler et al. 2014), and to domain-specific aptitudes (Golestani et al. 2011; Reiterer et al. 2011; Seither-Preisler et al. 2014).

Using structural magnetic resonance imaging (MRI), we recently showed brain structural, grey matter differences between phonetics experts and non-expert individuals in bilateral auditory and left inferior frontal (IFG) brain regions (Golestani et al. 2011). In particular, we found larger volumes of the transverse temporal gyri of the auditory cortex bilaterally in phoneticians compared to non-experts, and also a larger grey matter volume of the left pars opercularis in the experts. In addition, we found that in the phoneticians group, the grey matter volume of this same left inferior frontal region correlated positively with the amount of transcription training, suggesting that extensive training with speech analysis and phonetic segmentation results in structural plasticity of a brain region known to functionally subserve phonetic processing (Nixon et al. 2004; Zatorre et al. 1996; Burton et al. 2000; Gough et al. 2005). The larger volumes in auditory cortex for phoneticians compared to non-phoneticians is consistent with a previous study that found differences in the volume of the left auditory cortex, along with differences in parietal lobe volumes in fast compared to slow phonetic learners (Golestani et al. 2007), convergent with the results of a similar study in an independent group of participants (Golestani et al. 2002). Together this prior literature highlights structural differences in fronto-temporo-parietal systems related to skill and expertise in speech sound perception, with additional differences in the left insula noted in individuals who are skilled at pronouncing foreign speech sounds (Golestani and Pallier 2007).

**Fig. 1:**
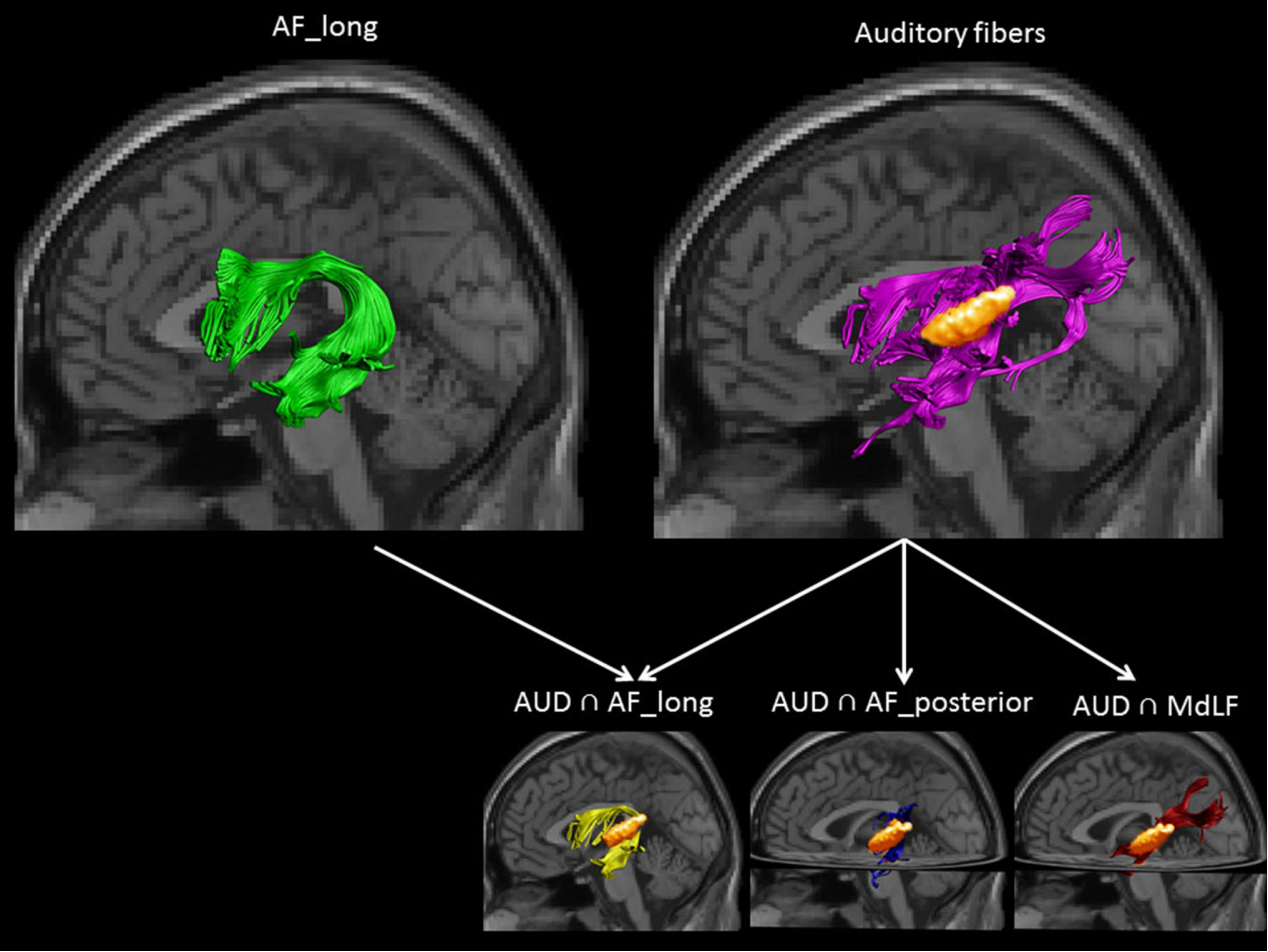
Example of the delineated white matter bundles in one representative control participant: the *upper left panel* shows AF_long, depicted in *green*, and the *upper right panel* shows the auditory fibers, depicted in *purple*, and the auditory ROI, depicted in *orange*. The *lower panel* shows the subdivisions of auditory fibers into (1) ones belonging to AF_long, depicted in *yellow*, (2) ones belonging to AF_posterior, depicted in *blue*, and (3) ones belonging to the middle longitudinal fasciculus (MdLF), depicted in *red*

Less is known about the white matter properties of the language system in phonetics experts. Previous diffusion MRI (DTI) studies have shown that the long segment of arcuate fasciculus (AF_long), a white matter tract connecting Broca’s area to the temporal cortex, plays a key role in language (for a review see Dick and Trembley 2012) (Fig. 1, upper left panel). In light of the proposed distinction between the dorsal audio-motor interface and the ventral meaning integration interface (Rodriguez-Fornells et al. 2009; Lopez-Barroso et al. 2013; Aboitiz 2012; Hickok and Poeppel 2007), it can be expected that especially pathways which form part of the dorsal system such as AF_long might be different in phonetics experts. Indirect support for this hypothesis is also given by the fact that AF_long connects, among other regions, the auditory and frontal regions for which Golestani and colleagues (2011) reported structural grey matter differences in phoneticians. However, AF_long has not been investigated in phoneticians, and especially the fibers within this language tract that specifically connect with the auditory cortex have not been investigated in this expert group (Fig. 1, lower left panel). With regards to other white matter fibers that connect to and arise from the auditory cortex, two subdivisions project posteriorly to parietal regions, one via the posterior segment of the arcuate fasciculus (AF_posterior) (Thiebaut de Schotten et al. 2011) and one via the middle longitudinal fasciculus (MdLF) (Makris et al. 2009) (Fig. 1, lower middle and right panels). Despite the suggested roles of AF_posterior (Thiebaut de Schotten et al. 2011; Vandermosten et al. 2012) and MdLF [(Saur et al. 2008); but see (De Witt Hamer et al. 2011)] in language processing, their specific connections to the auditory cortex have not been investigated.

In the present study, we used DTI to examine white matter differences between phoneticians and non-expert individuals, and training-related differences in the expert group, in (1) the AF_long (Fig. 1, upper left panel), and in (2) auditory fibers, i.e. all the fibers arising from the auditory cortex (Fig. 1, upper right panel). Although there is some overlap between the AF_long per se (i.e., in its entirety) and auditory fibers, the delineation of both of these provides complementary information. Namely, the AF_long is a well-validated tract but projects to and from several temporal regions (i.e. not exclusively the auditory cortex) and the frontal cortex. In contrast, auditory fibers have a higher specificity for the auditory cortex, but they belong to multiple, distinct white matter language tracts, each of which has its own course and projection points. In order to pinpoint the locus of group and training effects within the auditory fibers, we therefore also explored white matter organisation in three subdivisions of these auditory fibers (1) auditory fibers belonging to AF_long (AUD ∩ AF_long), (2) auditory fibers belonging to AF_posterior (AUD ∩ AF_posterior), and (3) auditory fibers belonging to MdLF (AUD ∩ MdLF) (Fig. 1, lower panel). The latter analyses were exploratory since these auditory fiber subdivisions could not be delineated in all subjects, and since the auditory fibers also contain fibers that do not belong to any of these three subdivisions. Group and training-related differences in the fractional anisotropy (FA) of these tracts were evaluated (see Supplementary Information for analyses on axial and radial diffusivity).

## Methods

### Participants

In this study, 33 right-handed adults participated, all screened for neurological and psychiatric problems. Seventeen participants were phoneticians (11 men) and they reported 1 to 4 years of formal training in phonetic transcription (*M* = 2.1, SD = 0.86), and 16 participants (6 men) were non-expert controls (i.e. no formal training in phonetics). The phonetician sample is exactly the same as described in Golestani et al. (2011), and the control sample largely overlapped. Four controls were not included relative to Golestani et al. (2011) due to missing DTI-data (3 participants) and unsuccessful DTI-acquisition (1 participant). In order to balance the number of participants across both groups, 4 new control participants were included in the present study. The selected phoneticians and controls did not show significant differences in gender (Fisher’s exact test: *p* = 0.17) nor in age (controls: *M* = 33.3, SD = 7.4; phoneticians: *M* = 39.8, SD = 13.2; *t*(31) = −1.72, *p* = 0.10*).* However, there were group differences in multilingual experience because the phoneticians were more multilingual than were the controls. Four of them were early bilinguals, and the phoneticians had received formal language instruction in up to 10 languages (mean number of languages 5.6 ± 2.1), whereas the controls had received formal language instruction in up to four languages. Our analyses therefore also investigated how any differences between phoneticians and controls, or the effect of years of transcription experience in the phoneticians might be influenced by the multilingual language experience of the phoneticians.

### DTI acquisition

Participants were imaged on a 1.5-T scanner (Siemens Sonata) with a phased-array head coil. Echo-planar images were acquired in the axial plane: 68 volumes with different directions of the diffusion encoding gradients and different *b* values (*b* = 100 s/mm^2^ during the first 7 volumes and *b* = 1000 s/mm^2^ for the remaining 61 volumes). Per volume, 60 axial slices were acquired with an isotropic resolution of 2.3 mm, and with FOV = 220 × 156, inter-slice temporal separation = 155 ms, TE = 90 ms, and flip angle = 90°. Cardiac gating was employed. In total the DTI scan lasted 25 min.

### DTI preprocessing

DTI preprocessing was performed by using the software program ExploreDTI (Leemans et al. 2009). The pre-processing steps consisted of visual quality assurance and rigorous motion and eddy current correction with the required reorientation of the *b* matrix (Leemans and Jones 2009), and an iterative nonlinear tensor estimation process to generate maps of FA. The individual datasets were non-rigidly normalized to MNI (Montreal Neurological Institute) space. Next, whole brain tractography was performed for each normalized DTI dataset using a step-size of 2 mm, a fractional anisotropy (FA) threshold of 0.2 to initiate and continue tracking, an angle threshold of 30°, and a fiber length range of 50–500 mm.

### DTI fiber tracking

First, we delineated AF_long, which is the fronto-temporal segment of the arcuate fasciculus (Fig. 1, upper left panel). For more details on ROI-placing see paper by Vandermosten and colleagues (2012). Second, we delineated all fibers passing through the left and right auditory cortex, with the latter defined as a combination of Heschl’s gyrus and the planum temporale according to the Harvard-Oxford atlas (25 % probability threshold) (Fig. 1, upper right panel). These auditory fibers do not correspond to a unique, well-described tract, according to diffusion MRI atlases and post-mortem research (Catani and Thiebaut de Schotten 2012; Wakana 2007). In order to better understand the locus of group and training effects within the auditory fibers, we also examined subdivisions of the auditory fibers, specifically in relation to three different validated language tracts (Fig. 1, lower panel). Specifically, we examined: (1) auditory fibers belonging to the AF_long (AUD ∩ AF_long), (2) auditory fibers belonging to the posterior parieto-temporal segment of the arcuate fasciculus (AUD ∩ AF_posterior), and (3) auditory fibers belonging to the middle longitudinal fasciculus (AUD ∩ MdLF). We delineated the AUD ∩ AF_long and AUD ∩ AF_posterior by delineating the two segments of the arcuate fasciculus in line with validated white matter atlases (Catani and Thiebaut de Schotten 2012), and by then selecting the subdivision of fibers that intersected with the auditory fibers. In order to delineate AUD ∩ MdLF, we first placed seed ROIs to segment the stem portion of the MdLF on five consecutive coronal slices of the FA color-coded maps (as described by Makris et al. 2009), and then we selected the fibers that overlapped with the auditory fibers. Although the existence of the MdLF is debated (Dick and Trembley 2012), in all but the right hemisphere of one subject we observed anterior–posteriorly oriented auditory fibers that were located within the white matter of superior temporal gyrus (STG). These fibers correspond to the MdLF, as described in previous DTI studies (Makris et al. 2009; de Champfleur et al. 2013).The number of missing data was substantial for some of the other auditory fiber subdivisions (see *N* values in Table 1), and although the number of missing values per tract did not significantly differ between groups (Fisher exact test: *p* > 0.34), results should be interpreted with caution. When it was possible to reconstruct these, we extracted the mean fractional anisotropy (FA) for each white matter bundle (AF_long, the auditory fibers and its three subsets: AUD ∩ AF_long, AUD ∩ AF_posterior, and AUD ∩ MdLF) in the left and right hemisphere, for each subject. Summary statistics per group are provided in Table 1. Additional analyses on axial and radial diffusivity are provided in the SI.

**Table 1:** Summary statistics of FA in the delineated set of fibers (AF_long, the auditory fibers and the three auditory fiber subdivisions) for phoneticians and controls

Fractional anisotropy (FA)	Phoneticians		Controls	
	Mean (SD)	*N* (total = 17)	Mean (SD)	*N* (total 16)
AF_long*				
Left	0.532 (0.019)	17	0.535 (0.016)	16
Right	0.511 (0.028)	17	0.519 (0.021)	15
Auditory fibers*				
Left	0.456 (0.019)	17	0.468 (0.022)	16
Right	0.445 (0.023)	17	0.472 (0.023)	16
Subdivisions auditory fibers				
Left AUD ∩ AF_long*	0.469 (0.029)	16	0.494 (0.039)	15
Right AUD ∩ AF_long	0.449 (0.038)	11	0.449 (0.036)	8
Left AUD ∩ AF_posterior	0.448 (0.041)	13	0.464 (0.029)	15
Right AUD ∩ AF_posterior*	0.436 (0.037)	13	0.481 (0.024)	12
Left AUD ∩ MdLF	0.456 (0.020)	17	0.468 (0.022)	16
Right AUD ∩ MdLF (*)	0.448 (0.026)	16	0.470 (0.029)	16

Note: Results of the auditory fiber subdivisions should be interpreted with caution since for some subdivisions there is a high number of missing values, and also since post hoc tests were not corrected for multiple comparisons

(*) *p* < 0.10, * *p* < 0.05

### Statistics

FA for the delineated bilateral white matter bundles (i.e. AF_long, auditory fibers and its three subdivisions) were analyzed using Mixed Models (Littell et al. 2006). More specifically, for AF_long and the auditory fibers, FA-values were analyzed by means of 2 (group: phoneticians vs. controls) × 2 (hemisphere: left vs. right) full factorial models. The variable group (i.e. phoneticians and controls) was included as a between-subjects variable, hemisphere as a within-subjects variable, subject as a random variable, and mean FA across the whole brain as a covariate (to control for overall FA differences which might be due to motion, age, gender, etc.). For the subdivisions of auditory fibers, the variable ‘subdivision’ (i.e. AUD ∩ AF_long, AUD ∩ AF_posterior, AUD ∩ MdLF) was included as an additional within-subjects variable in the factorial analyses. The use of mixed model analyses has some important advantages over (paired) t-tests (Verbeke and Lesaffre 1997): (a) it is much more robust when analyzing semi-normally distributed data (a trend for semi-normally distributed data was found for the residuals of AF_long, *p* = 0.09, tested using a Shapiro–Wilk test), (b) it allowed us to account for the fact that specific pairs of fibers from the left and right hemispheres belong to the same subject, (c) it allows covariates to be incorporated, and (d) it can handle missing or non-balanced data (see Table 1 for the number of missing data per delineated tract). In order to test for training-related differences in FA within the phoneticians group along the delineated fibers of interest, we also ran Spearman correlations, which are suited for small sample sizes.

## Results

Mean FA values for each of the investigated set of fibers for both groups are presented in Table 1. For AF_long, there was no significant main effect of group (*F*(1, 30) = 0.24, *p* = 0.630) nor was there a significant interaction (*F*(1, 30) = 0.47, *p* = 0.497), but consistent with its role in language processing and with normative reports (Thiebaut de Schotten et al. 2011), there was a main effect of hemisphere, with higher FA on the left compared to the right (*F*(1, 29) = 20.76, *p* < 0.001).

**Fig. 2:**
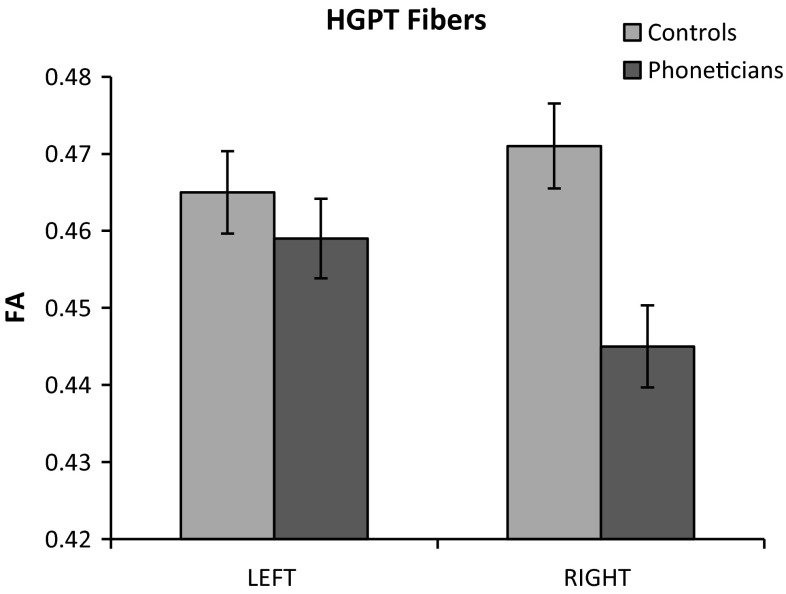
Average FA for the phoneticians (*dark grey*) and controls (*light grey*) in the auditory fibers. *Error bars* indicate plus and minus one standard error of the mean per group

For the auditory fibers, there was a main effect of group, (*F*(1, 30) = 8.19, *p* = 0.008), with lower FA in the phoneticians group. The effect of hemisphere was not significant (*F*(1, 30) = 0.71, *p* = 0.407), nor was there a significant group by hemisphere interaction (*F*(1, 30) = 3.00, *p* = 0.094). The average FA (and error bars) for the left and right auditory fibers for both groups are depicted in Fig. 2. The analysis on the three subdivisions of auditory fibers revealed a significant main effect of group (*F*(1,124) = 10.34, *p* = 0.002) with lower FA in the phoneticians, in line with the group difference observed for the auditory fibers. This group effect was not specific to the left or right hemisphere (group × hemisphere: *F*(1, 124) = 0.40, *p* = 0.529) nor to one particular auditory subdivision of fibers (group × subdivision: *F*(2, 124) = 0.90, *p* = 0.408). However, there was a significant three-way, group × subdivision × hemisphere interaction (*F*(4, 124) = 2.95; *p* = 0.023). Post-hoc analyses on this interaction showed that in the left hemisphere, the group difference was driven by auditory fibers belonging to AF_long (*t* = 2.18, *p* = 0.031), and not by auditory fibers belonging to AF_post (*t* = 1.29, *p* = 0.201) nor to MdLF (*t* = 1.03, *p* = 0.305). Yet in the right hemisphere the pattern was opposite, with the group difference driven by the auditory fibers belonging to AF_posterior (*t* = 3.4, *p* = 0.001) and to some extent to MdLF (*t* = 1.83, *p* = 0.069), but not by the auditory fibers belonging to AF_long (*t* = 0.41, *p* = 0.686). As displayed in Table 1, phoneticians had a lower FA compared to controls. Note, however, the high number of missing fibers in right AUD ∩ AF_posterior and in right AUD ∩ AF_long, and note that although right AUD ∩ AF_posterior survived Bonferroni correction, the left AUD ∩ AF_long did not. Therefore, results on the auditory subdivisions should be regarded as exploratory and should be interpreted with caution.

**Fig. 3:**
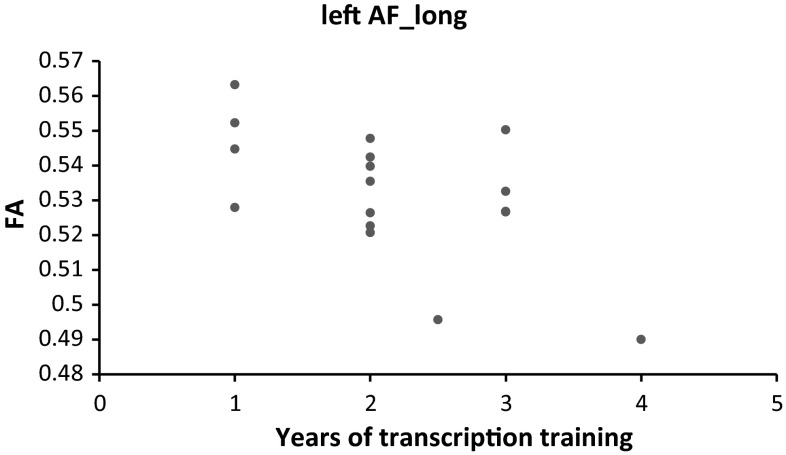
*Scatter plot* showing the relationship between FA in the left fronto-temporal segment of the arcuate fasciculus (AF_long) and years of transcription training in the phoneticians group

In order to test if the FA along any of the bundles of interest (AF_long, auditory fibers and its subdivisions) predicts the years of phonetic training in the expert group, we ran Spearman correlations. Results revealed that only FA in the left AF_long predicts years of phonetic training in the experts (*r* = −0.498, *p* = 0.0421). The direction of the relationship between FA in this tract and phonetic transcription training is negative, with more years of training being associated with lower FA along this tract (see Fig. 3). This relationship remains present when taking into account individual differences in the multilingual language experience of the phoneticians (*r* = 0.515, *p* = 0.041) (for more details on the language background measure see Golestani et al. 2011).

## Discussion

We find evidence for reduced FA in the white matter fibers arising from the bilateral auditory cortices in the phoneticians compared to controls. This result converges with our previous finding, from mostly the same participants, that the transverse gyri were larger bilaterally in the phoneticians compared to the non-experts (Golestani et al. 2011). This group difference may have arisen from training-related plasticity in the auditory cortex in this expert group, and/or from pre-existing structural differences in the phoneticians compared to the non-expert individuals. We also found evidence, within the phoneticians group, that FA along the left fronto-temporal segment of arcuate fasciculus (i.e. AF-long) negatively predicts the years of phonetic training, with lower FA values along this tract predicting more training. White matter atlases show that this fronto-temporal segment of the AF projects to IFG regions including the pars opercularis (Catani and Thiebaut de Schotten 2012). The effect of phonetic training that we observed in the left fronto-temporal segment converges with our previous findings that grey matter volume in the left pars opercularis increases with phonetic training (Golestani et al. 2011). It is also consistent with the known role of this region in phonological processing (Nixon et al. 2004; Zatorre et al. 1996; Burton et al. 2000; Gough et al. 2005). Based on the training-related differences in the left AF_long and on the previous finding of a group difference in the volume of the left pars opercularis (Golestani et al. 2011), we expected to also find a group difference in the left AF_long. We did not observe a group difference in the AF_long per se; however, a group difference was present when specifically examining the fibers of left AF_long that connect with the auditory cortex. This result, taken together with the previous grey matter volume findings (Golestani et al. 2011), suggests that the previously observed left pars opercularis volume difference arises from differences in the anatomy of this region in relation to auditory cortex structure (and function), and that left fronto-temporal fibers sustain speech sound analysis and segmentation in phoneticians.

Our findings demonstrate group and training-related differences within the dorsal audio-motor interface pathway connecting auditory, frontal and parietal regions. This pathway is known to be involved in mapping sounds onto articulatory-based representations (Rodriguez-Fornells et al. 2009; Hickok and Poeppel 2007), and is also known to be especially relevant for phonological processing and phonological working memory (Aboitiz 2012). Exploratory analyses on the subdivisions of auditory fibers indicated that the left hemispheric group differences were mainly driven by fronto-temporal fibers (i.e. AUD ∩ AF_long), as discussed above, whereas that right hemispheric differences were mainly driven by parieto-temporal fibers (i.e. AUD ∩ AF_posterior). This latter finding shows some convergence with previous findings of differences in parietal cortex volume asymmetries in faster compared to slower phonetic learners (Golestani et al. 2002, 2007), and might be related to more general speech (Vandermosten et al. 2012) and language learning mechanisms (Golestani and Zatorre 2004; Lopez-Barroso et al. 2013), but should be interpreted with caution due to the high number of missing data points.

The direction of both the group and training-related differences, with lower FA in the experts compared to controls and in the experts as a function of training, are opposite to what might be expected when interpreting FA as a quantitative biomarker of white matter ‘integrity’. However, equating FA with an index of white matter integrity is an oversimplified interpretation (Jones et al. 2013), and contrasting effects of training on FA are also apparent in previous studies, with some showing higher FA in functionally relevant brain regions in relation to training or to skill (Bengtsson et al. 2005; Sampaio-Baptista et al. 2013; Schlegel et al. 2012; Tomassini et al. 2011), and others showing the opposite, i.e. lower FA as a function of training, learning skill or expertise (Bengtsson et al. 2005; Roberts et al. 2013; Elmer et al. 2011; Imfeld et al. 2009; Tuch et al. 2005; Schmithorst and Wilke 2002; Steele et al. 2012; Wegman et al. 2014; Yeatman et al. 2012). Our results also fit nicely with a DTI study in which FA in the left arcuate fasciculus, and especially in the fibers that connect STG and IFG (i.e. AF_long), was negatively related with the years of vocal training in professional singers and in individuals training to become professional singers (Halwani et al. 2011).

The contrasting effects of training and expertise on FA can be explained by appreciating that FA reflects a composite of microscopic and macroscopic factors (Mori 2007). According to this model, lower FA related to years of phonetic expertise or training, as observed in auditory and fronto-temporal fibers in the current study, could therefore arise from (1) lower fiber density, as a consequence of training-related pruning, (2) greater fiber complexity, or (3) less myelination as a result of either better tuned connections within specialized speech networks or less need for rapid neural transmission in some parts of the network.

Recent findings on neural fibers from both animals and humans indicate that axon properties, rather than myelination, play a predominant role in anisotropy (for reviews see (Beaullieu 2009; Paus 2010). This implies that lower FA in the phoneticians is more likely to be explained by processes such as pruning and fiber complexity than lower myelination. Myelin has nonetheless also been shown to influence FA, with studies on genetically modified species that lack myelin showing that FA values are, on average, 15 % less in the dysmyelination models (Beaulieu 2009). It should be noted, however, that myelin volume and axon density are often confounded in studies where myelinated versus non-myelinated axons are compared, rather than comparing axons with varying degrees of myelination (Beaulieu 2009). Based on animal studies (e.g. Song et al. 2002), quantifying axial and radial diffusivity in addition to FA is generally seen as an indirect way to provide more specific information on axon and myelin properties, respectively. Although this approach is controversial (Wheeler-Kingshott et al. 2009), we examined these two diffusion indices (see SI), and neither specifically contributes to the observed FA findings.

In conclusion, our DTI findings converge with previously published structural imaging work in showing differences in phonetics experts compared to non-experts in the white matter microstructure of fibers connecting auditory regions, and of the arcuate fasciculus, connecting portions of the left IFG to the temporal cortex, as a function of training in the experts. These latter white matter differences could reflect expertise-related pruning or increased complexity of white matter fibers connecting fronto-temporal functional hubs that are important for phonetic processing. Further longitudinal work would serve to elucidate the dynamics and direction of grey and white matter plasticity in this expert group.

## Electronic supplementary material

Below is the link to the electronic supplementary material.

Supplementary material 2 (DOCX 18 kb)
