# Supplementary material for: Plasticity of white matter connectivity in phonetics experts
Source: Brain Struct Funct. 2015 Sep 19;221(7):3825–33. doi: 10.1007/s00429-015-1114-8 (PMC5009160; doi:10.1007/s00429-015-1114-8)
Supplement: Supplementary file 1 — Supplementary material 2 (DOCX 18 kb) [file 429_2015_1114_MOESM1_ESM.docx]

# Supplementary Information

## Microscopic aspects

In order to aid in the interpretation of FA-findings, we also examined radial diffusivity, which is the diffusivity in directions perpendicular to the principal axis of diffusion (i.e. λ2 & λ3), as well as axial diffusivity, which is the diffusivity along the principal axis (i.e. λ1). Based on longitudinal scanning experiments in animals (Harsan et al., 2006; Song et al., 2002, 2003; 2005; Sun et al., 2006; 2008), radial and axial diffusivity have often been used in literature as a marker for myelin and axonal properties, respectively. It should be noted, however, that the voxel size greatly exceeds the resolution of microscopic factors and that diffusion indices are mainly determined by macroscopic rather than by microscopic factors such as myelination (for a review see Beaulieu, 2009). Therefore, inferences about the association between microscopic processes and axial and radial diffusivity should be made with the same extreme caution as for FA (Wheeler-Kingshott et al., 2009).

## With regard to the group difference for FA in the auditory fibers reported in the main text, we ran additional mixed models analyses to determine whether this effect was specifically due to radial or axial diffusivity differences. Radial and axial diffusivity indices were analyzed within a 2 (Group: phoneticians vs. controls) x 2 (Hemisphere: left vs. right) x 2 (diffusivity: axial vs. radial) full-factorial model. The variable group (i.e. phoneticians and controls) was included as a between-subject variable, hemisphere and diffusivity as within-subject variable, subject as a random variable, and mean FA across the brain as a covariate. A main effect of group was found [F (1, 92) = 4.92, p = .029], reflecting the group difference in FA, however, no interactions between group and diffusivity were found (group x diffusivity: F (1, 92) = 0.93, p = .337; group x diffusivity x hemisphere: F (1, 92) = 0.79, p = .378). With regard to the observed correlation between FA and AF_long reported in the main text, here we ran regression analyses with radial and axial diffusivity of left AF_long as predictors. None of the two had a unique contribution in predicting years of phonetic training (axial: *t*(1) = -1.27, *p* = .226; radial: *t*(1)=1.97, *p* = .070). Thus, overall, the mixed models and regression analyses show that the significant effects observed for FA are not specifically driven by differences in radial or axial diffusivity, but rather by a combination of both.
